# Contraceptive Use Measured in a National Population–Based Approach: Cross-Sectional Study of Administrative Versus Survey Data

**DOI:** 10.2196/45030

**Published:** 2024-07-22

**Authors:** Juliette Congy, Delphine Rahib, Céline Leroy, Jean Bouyer, Elise de La Rochebrochard

**Affiliations:** 1 Sexual and Reproductive Health and Rights Unit Institut National d'Etudes Démographiques Aubervilliers France; 2 Sexual Health Unit Santé Publique France Saint-Maurice France; 3 Agence Régionale de Santé Caen France; 4 Centre de Recherche en Epidémiologie et Santé des Populations Institut national de la santé et de la recherche médicale Université de Versailles Saint-Quentin-en-Yvelines, Université Paris-Saclay Villejuif France

**Keywords:** contraception, administrative data, health data, implant, oral contraceptives, intrauterine device, IUD, contraceptive prevalence, contraceptive, birth control, monitoring, public health issue, population-based survey, prevalence

## Abstract

**Background:**

Prescribed contraception is used worldwide by over 400 million women of reproductive age. Monitoring contraceptive use is a major public health issue that usually relies on population-based surveys. However, these surveys are conducted on average every 6 years and do not allow close follow-up of contraceptive use. Moreover, their sample size is often too limited for the study of specific population subgroups such as people with low income. Health administrative data could be an innovative and less costly source to study contraceptive use.

**Objective:**

We aimed to explore the potential of health administrative data to study prescribed contraceptive use and compare these data with observations based on survey data.

**Methods:**

We selected all women aged 15-49 years, covered by French health insurance and living in France, in the health administrative database, which covers 98% of the resident population (n=14,788,124), and in the last French population–based representative survey, the Health Barometer Survey, conducted in 2016 (n=4285). In health administrative data, contraceptive use was recorded with detailed information on the product delivered, whereas in the survey, it was self-declared by the women. In both sources, the prevalence of contraceptive use was estimated globally for all prescribed contraceptives and by type of contraceptive: oral contraceptives, intrauterine devices (IUDs), and implants. Prevalences were analyzed by age.

**Results:**

There were more low-income women in health administrative data than in the population-based survey (1,576,066/14,770,256, 11% vs 188/4285, 7%, respectively; *P*<.001). In health administrative data, 47.6% (7034,710/14,770,256; 95% CI 47.6%-47.7%) of women aged 15-49 years used a prescribed contraceptive versus 50.5% (2297/4285; 95% CI 49.1%-52.0%) in the population-based survey. Considering prevalences by the type of contraceptive in health administrative data versus survey data, they were 26.9% (95% CI 26.9%-26.9%) versus 27.7% (95% CI 26.4%-29.0%) for oral contraceptives, 17.7% (95% CI 17.7%-17.8%) versus 19.6% (95% CI 18.5%-20.8%) for IUDs, and 3% (95% CI 3.0%-3.0%) versus 3.2% (95% CI 2.7%-3.7%) for implants. In both sources, the same overall tendency in prevalence was observed for these 3 contraceptives. Implants remained little used at all ages, oral contraceptives were highly used among young women, whereas IUD use was low among young women.

**Conclusions:**

Compared with survey data, health administrative data exhibited the same overall tendencies for oral contraceptives, IUDs, and implants. One of the main strengths of health administrative data is the high quality of information on contraceptive use and the large number of observations, allowing studies of subgroups of population. Health administrative data therefore appear as a promising new source to monitor contraception in a population-based approach. They could open new perspectives for research and be a valuable new asset to guide public policies on reproductive and sexual health.

## Introduction

Prescribed contraception is used worldwide by more than 400 million women of reproductive age [1]. It must be monitored closely to implement public health policy or decisions and ensure rapid response to societal, media, and medical events. For example, after the media scare regarding increased cardiovascular risks for women using third- or fourth-generation pills, a large number of women abruptly stopped using these pills [2-5]. More recently, with the COVID-19 pandemic, the disruption of some supply chains threatened the availability of these contraceptives [6] and a possible increased risk of thromboembolism when contracting COVID-19 while using hormonal contraceptives was investigated [7,8]. Contraceptive use also needs to be monitored to study its long-term effects, particularly in view of recent studies looking into an association between hormonal contraceptive use and a wide range of indicators such as ectopic pregnancy, pancreatic cancer, and depression [9-11].

Contraceptive use is monitored worldwide by the United Nations Population Division, whose findings are regularly published in World Contraceptive Use [1]. These statistics are based on national population–based surveys including the Demographic and Health Surveys [12] and the Fertility and Family Surveys [13]. However, such surveys are time-consuming and costly, resulting in 2 major limitations. First, they are conducted only about every 6 years and so do not allow close follow-up of contraceptive use over time [14,15]. Second, their sample size is restricted by the cost of the survey, which leads to serious limitations of statistical power when aiming to explore specific subgroups (such as low-income people who are often hard to reach in population-based surveys) or lesser-used contraceptives (such as implants). It would therefore be useful to examine new sources to monitor contraceptive use in a national population–based approach.

Health administrative data could provide an innovative and low-cost source to monitor the use of prescribed contraceptives. Theoretically, these data could provide comprehensive, time-continuous information on prescribed contraception in very large samples that include all the resident population covered by health insurance [16]. Considering the limitations of population-based surveys, health administrative data could be a very powerful alternative to monitor contraceptive use, even if neither source can be considered as a gold standard.

We aimed to explore the potential of health administrative data to study prescribed contraceptive use and compare these data with observations based on surveys.

## Methods

### Administrative and Survey Sources

The French health administrative database records data from the national health insurance system, which covers health care based on reimbursements determined at the national level. It includes 98% of the resident population, whether of French nationality or not. These data have been presented in detail elsewhere [16,17]. They provide information on all health care reimbursements including medicines, medical devices, medical procedures, laboratory tests, and hospital admissions. Dates of prescription and delivery are recorded as well as some information on the patient: age, sex, and place of residence. Persons with low income are identified through their registration (yes or no) with specific health care insurance for low-income people. This specific insurance is granted to persons below the poverty line, that is, with an income less than 50% of the median income.

The last French population–based survey on contraceptive use, the Health Barometer Survey, was conducted in 2016 [18,19]. Through a 2-level survey (household and then individual), a representative sample was recruited, including 15,216 people aged 15-75 years living in metropolitan France and speaking French. Data were collected through phone interviews based on a 37-minute questionnaire on sexual health.

### Study Population

The population studied comprised all women aged between 15 and 49 years in 2016, covered by French health insurance, and living in mainland France, based on both French health insurance data (n=14,788,124) and survey data (n=4285).

### Outcome

Contraceptive use was defined as the use (yes or no) of a prescribed contraceptive by the woman. Three types of contraceptives were considered: oral contraceptives, intrauterine devices (IUDs), and implants.

In the French health administrative database, prescribed contraceptives are recorded automatically at purchase at the pharmacy, which directly transfers data to the national system with detailed information on the product (Multimedia Appendix 1). The very few contraceptives delivered through community structures are not recorded in the database. Data include only reimbursed contraceptives (oral contraceptives, except third- and fourth-generation pills that are not reimbursed in France; IUDs; and implants). We studied the population that was using contraception on December 31, 2016. Women were considered to be using contraception if their last prescribed contraceptive purchased had a recommended duration of use still ongoing at that date. For example, if the last prescribed contraceptive was an implant, and it was bought less than 3 years previously (the recommended duration of use), it was considered as still ongoing on December 31, 2016.

In the French survey, contraceptive use was measured based on response to the following question: “Currently, do you, or your partner, use a method to avoid pregnancy, including natural methods, and if so, which one?” Sixteen different methods were listed: pill, IUD, implant, diaphragm, patch, ring, male condom, female condom, withdrawal, avoidance of intercourse on the days most at risk of pregnancy, spermicides (creams, ovules, and sponges), hormonal injection, morning-after pill, abstinence, tubal ligation, and vasectomy. These methods were collected whether prescribed and reimbursed (such as the IUD or the implant), prescribed and not reimbursed (such as the patch or the ring), or nonprescribed (such as the withdrawal or natural methods). If the method was “the pill,” no information was collected on the generation of the pill. This category therefore included both first- and second-generation pills (reimbursed in France) and third- and fourth-generation pills (not reimbursed). In this study, only contraceptive methods included in the French health administrative database were considered, and the type of contraceptive used was coded as a 3-group variable: oral contraceptive (pill), IUD, or implant. Contraceptive use was collected for all women regardless of their relationship status, taking into consideration the possibility that they used contraception outside of marriage or partnerships. To avoid unnecessary questions, it was presumed that women who were not likely to become pregnant did not use contraception (pregnant women, women who did not have sexual intercourse, and those who had sexual intercourse only with women). These women were directly recoded as not using any contraception.

### Statistical Analysis

In the French health administrative data and in the population-based survey, the prevalence of contraceptive use was calculated among all women aged 15-49 years (including unmarried women, women who were not in a relationship, pregnant women, women who did not have sexual intercourse, and those who had sexual intercourse only with women). The prevalence was estimated for all prescribed contraceptives and by type of contraceptive (oral contraceptive, IUD, and implant). The 2 sources were not comparable regarding the measurement of oral contraceptive use, as French health administrative data included only first- and second-generation pills, whereas the population-based survey included indiscriminately first-, second-, third-, and fourth-generation pills. To obtain comparable estimates in both sources, the prevalence of oral contraceptive use in health administrative data was adjusted by a factor of *1.2195 = (1 + 0.18 / 0.82)*, as third- and fourth-generation pills account for 18% (519,548/2,906,112) of estrogen-progestin pill sales in France (see Multimedia Appendix 2 for details). In the survey, all estimates were weighted in order to take into account the 2-level design of the study and correction for undercoverage. We carried out analyses using SAS (version 9.4; SAS Institute).

### Ethical Considerations

The French health administrative database is accessible through the French Système National des Données de Santé (SNDS; National Decree 2016-316, October 13, 2016). French law allows the use of personal data from the SNDS for health research without requiring the express or written consent of individual subjects (deliberation 2016-263, July 21, 2016). Participants did not receive any compensation. The French National Consultative Ethics Committee analyzed ethical issues of big data and approved the use of personal data without requiring the individual’s consent, considering that their use for public health research corresponds to an ethical principle of solidarity and fraternity (committee opinion 130). All SNDS data are pseudonymized. Two authors (JC and ELR) took SNDS training courses and obtained permission to access data remotely for the duration of the present project under the legal responsibility of Institut National d’Etudes Démographiques, which has permanent access to the SNDS (National Decree 2016-1871, December 26, 2016). This research was approved by the Institut National d’Etudes Démographiques Data Protection Officer (reference 2019-DPD-0013). The last French population–based survey on contraceptive use, the Health Barometer Survey, was approved by the National Data Protection Authority (reference 915589).

## Results

In health administrative data, women were almost evenly distributed across age groups (Table 1). In comparison, in the survey data, younger women (<25 years) were underrepresented, and older women (≥40 years) were overrepresented. In health administrative data, 11% (1,576,066/14,770,256) were women with low income, whereas this proportion was 7% (188/4285) in the survey (*P*<.001).

Among women aged 15-49 years, the prevalence of prescribed contraception was 47.6% (95% CI 47.6%-47.7%) in health administrative data versus 50.5% (95% CI 49.1%-52.0%) in survey data (Table 2). More specifically, the prevalence of oral contraceptive use was 26.9% (95% CI 26.9%-26.9%) in health administrative data versus 27.7% (95% CI 26.4%-29.0%) in survey data. The prevalence of IUD use was 17.7% (95% CI 17.7%-17.8%) in health administrative data versus 19.6% (95% CI 18.5%-20.8%) in survey data. Finally, the prevalence of implants was 3% in both health administrative data and survey data (95% CI 3.0%-3.0% and 2.7%-3.7%, respectively).

**Table 1:** Characteristics of populations selected in health administrative data and population-based survey data for women aged 15-49 years in France 2016, cross-sectional study.

			Health administrative data (n=14,770,256), n (%)		Survey data^a^ (n=4285), n (%)	*P* value^b^	
**Age (years)**							.04
	15-19	1,943,189 (13.2)		352 (12.1)			
	20-24	1,923,509 (13)		488 (12.9)			
	25-29	2,093,726 (14.2)		588 (14.7)			
	30-34	2,173,256 (14.7)		647 (14.2)			
	35-39	2,185,765 (14.8)		694 (14.3)			
	40-44	2,196,345 (14.9)		741 (16.4)			
	45-49	2,254,465 (15.3)		775 (15.4)			
**Economic status^c^**							<.001
	Low-income women	1,576,066 (10.7)		188 (6.7)			
	Non–low-income women	13,194,190 (89.3)		4097 (93.3)			

^a^Weighted percentage.

^b^Chi-square test comparing distributions between health administrative data and weighted survey data.

^c^Economic status was identified through registration (yes or no) with specific health care insurance for low-income people. This specific insurance is granted to persons below the poverty line, that is, with an income less than 50% of the median income.

**Table 2:** Use of prescribed contraceptive by method in health administrative data and survey data for women aged 15-49 years in France 2016, cross-sectional study.

		Health administrative data (n=14,770,256), prevalence (%; 95% CI)	Survey data^a^ (n=4285), prevalence (%; 95% CI)	*P* value^b^
All contraceptives^c^		47.6 (47.6-47.7)	50.5 (49.1-52.0)	<.001
**Implants, IUDs^d^, and oral contraceptives**				<.001
	Implants	3.0 (3.0-3.0)	3.2 (2.7-3.7)	
	IUDs	17.7 (17.7-17.8)	19.6 (18.5-20.8)	
	Oral contraceptives	26.9 (26.9-26.9)	27.7 (26.4-29.0)	

^a^Weighted percentage.

^b^Chi-square test.

^c^All contraceptives include implants, IUDs, and oral contraceptives.

^d^IUD: intrauterine device.

**Figure 1:**
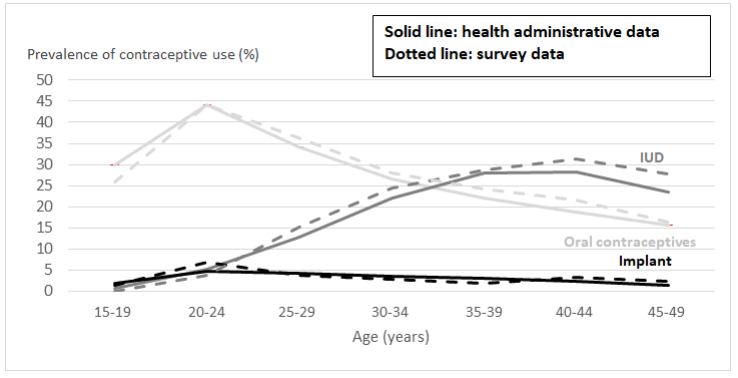
Use of prescribed contraceptive by method according to age, health administrative data and survey data for women aged 15-49 years in France 2016, cross-sectional study. IUD: intrauterine device.

The prevalence of use of the 3 types of contraceptives is detailed by age group in Figure 1 (also see Multimedia Appendix 3 for detailed statistics of this figure). The curves from the health administrative data (solid lines) and the survey data (dotted lines) followed a similar overall pattern and were very close for all 3 types of contraceptives. Implants were little used at all ages, with a peak around 5% (health administrative data: 89,210/1,923,509 and survey data: 29/488) among women aged 20-24 years. Oral contraceptives were widely used by young women, with a peak around 42% (health administrative data: 849,867/1,923,509 and survey data: 244/488) at age 20-24 years. Their prevalence then decreased to around 16% (health administrative data: 354,633/2,254,464 and survey data: 139/775) among women aged 45-49 years. On the contrary, IUD use was low among young women, being used by only around 7% (health administrative data: 99,244/2,254,464 and survey data: 16/488) of women aged 20-24 years. It increased with age to around 30% (health administrative data: 1,232,328/2,382,109 and survey data: 458/1435) in women aged 35-44 years and then showed a decrease to around 25% (health administrative data: 528,837/2,254,464 and survey data: 222/775).

## Discussion

### Principal Findings and Comparison With Prior Work

Using health administrative data, the prevalence of contraceptive use was estimated by type of contraceptive. In both health administrative and survey data, the same overall tendency in prevalence was observed across age groups for oral contraceptives, IUDs, and implants. Following a PubMed search and to the best of our knowledge, this study is the first to compare contraceptive use through both health administrative data and survey data. However, some authors have previously used health administrative data to estimate contraceptive use in Denmark, Finland, Iceland, Norway, Sweden, and the United Kingdom [20-26]. In all these countries, as in this French study, results were consistent: oral contraceptives were the most widely used method, followed by IUDs, whereas implants remained little used.

French health administrative data have already been highlighted as a new tool that provides high-quality data for pharmacoepidemiological study [17,27]. As shown in a paper comparing Nordic countries using their national health administrative data [24], these data can be particularly relevant for comparisons between countries. They can also be a valuable source for time-analysis studies. Health administrative data may be more homogeneous than data originating from different surveys. Moreover, they are statistically very powerful because of the large population from which they are drawn, allowing exploration of topics that are usually difficult to approach in surveys with limited sample size. For example, health administrative data would allow analysis of less-used contraceptives (such as implants) or could assess the impact of local policies on regional populations. Finally, health administrative data have already shown their value in guiding public health decisions, for example, in England, where they are used to support sexual health strategy [28].

### Limitations

One of the main strengths of health administrative data is the high quality of information on contraceptive use [16]. In this study, to allow comparisons, we limited the analysis to the 3 large classes of contraceptives that were measured in the survey: implants, IUDs, and oral contraceptives. Health administrative data would allow analysis of more detailed information, for example, the type of hormones used in the contraceptive. However, a limitation is that non-reimbursed medical contraception is not included in the health administrative database, so it is not possible to take condoms into account even though they are widely used, especially among young women [2]. In addition, nonmedical contraception, such as natural methods and the withdrawal method, is not included in the health administrative data. Depending on the study objective, these limitations may be insignificant or prohibitive and must be anticipated when considering the use of health administrative data to monitor contraceptive use. Another major strength of the French health administrative database is that it covers about 98% of the resident population [16], yielding a very large sample for analysis. With such data, the 95% CI is very narrow (with possibly the same value for the 2 limits of the interval when displayed with only 1 decimal), and statistical tests can be significant with a negligible gap between proportions. Thus, statistical differences should not be overinterpreted, and the gap between values should also always be considered.

With such a large sample, health administrative data allow low-income populations to be fully taken into account in research. Low-income populations are hard to reach in population-based surveys because they are known to respond less to surveys [29]. They may therefore be too few in number for statistical analysis in surveys. Moreover, considering their low participation rate, there is probably a selection bias in the recruitment of low-income populations. On the contrary, health administrative data offer a source that is nearly exhaustive and free of selection bias. The very large number of low-income women (1.6 million women aged 15-49 years) allowed accurate analysis of their contraceptive use by distinguishing the different types of contraceptives (oral contraceptives, IUDs, and implants).

In France, low-income persons benefit from 100% health coverage at no personal cost. In the health administrative health database, people with low income can be effectively identified through this special health coverage. This is a very strong advantage of French health administrative data compared with other national health databases such as the UK health care database, where disadvantage is not measured as an individual characteristic but only through a deprivation index [30,31].

A limitation of the French health administrative data is that it does not include non-reimbursed prescribed contraception, that is, third- and fourth-generation pills, patches, and vaginal rings. Patches and vaginal rings are rarely used in France (<1%) [18]. Third- and fourth-generation pills are more often used, so in this study, we adjusted oral contraceptive use to include these pills. Moreover, it cannot be ruled out that some contraceptives were purchased but never used or that a few women stopped using the contraceptive before the end of its recommended duration.

### Conclusions

In France, as in other countries, health administrative data are a promising new source for population-based monitoring of contraceptive use. They could open new perspectives for research and be a valuable asset to guide public policies on reproductive and sexual health.

## Appendix

Multimedia Appendix 1 Codes of prescribed contraceptives in the French health administrative database.

Multimedia Appendix 2 Estimation of contraceptive use prevalence in health administrative data.

Multimedia Appendix 3 Prescribed contraceptive use by type of contraceptive and age group in health administrative data and population-based survey data, women aged 15-49 years in France 2016 cross-sectional study.

## Abbreviations

IUD: intrauterine device
SNDS: Système National des Données de Santé

## Data Availability

The data sets generated and analyzed during this study are not publicly available for reasons of confidentiality but are available from the Health Data Hub repository on reasonable request. The data sets of the Health Barometer Survey analyzed during this study are available from author DR on reasonable request.

## References

[1] UN Population Division Data Portal: Interactive access to global demographic indicators. United Nations.

[2] Bajos N, Rouzaud-Cornabas M, Panjo H, Bohet A, Moreau C, The Fecond Team (2014). The French pill scare: towards a new contraceptive model?. Popul Soc.

[3] Balasch J (1997). The 'pill scare II' two years later. Eur J Contracept Reprod Health Care.

[4] Barnett J, Breakwell GM (2003). The social amplification of risk and the hazard sequence: the October 1995 oral contraceptive pill scare. Health Risk Soc.

[5] Lemaitre M, Lastennet G, Syr D, Emmerich J, Zureik M (2015). Impact of the 2013 French pill crisis on women's behaviour regarding contraception. Drugs Real World Outcomes.

[6] Aly J, Haeger KO, Christy AY, Johnson AM (2020). Contraception access during the COVID-19 pandemic. Contracept Reprod Med.

[7] Cohen MA, Edelman A, Paynter R, Henderson JT (2023). Risk of thromboembolism in patients with COVID-19 who are using hormonal contraception. Cochrane Database Syst Rev.

[8] Pascoal DB, de Araujo IM, Lopes LP, de Cruz CM (2021). Analysis of the role of female hormones during infection by COVID-19. Rev Bras Ginecol Obstet.

[9] Kopp-Kallner H, Linder M, Cesta CE, Chacón SS, Kieler H, Graner S (2022). Method of hormonal contraception and protective effects against ectopic pregnancy. Obstet Gynecol.

[10] Ilic M, Milicic B, Ilic I (2021). Association between oral contraceptive use and pancreatic cancer risk: a systematic review and meta-analysis. World J Gastroenterol.

[11] Gawronska J, Meads C, Smith L, Cao C, Wang N, Walker S (2024). Association of oral contraceptive pill use and depression among US women. J Affect Disord.

[12] Corsi DJ, Neuman M, Finlay JE, Subramanian SV (2012). Demographic and health surveys: a profile. Int J Epidemiol.

[13] Festy P, Prioux F (2002). An evaluation of the Fertility and Family Surveys project. United Nations.

[14] Moreau C, Bohet A, Hassoun D, Teboul M, Bajos N, FECOND Working Group (2013). Trends and determinants of use of long-acting reversible contraception use among young women in France: results from three national surveys conducted between 2000 and 2010. Fertil Steril.

[15] Mosher WD, Moreau C, Lantos H (2016). Trends and determinants of IUD use in the USA, 2002-2012. Hum Reprod.

[16] Tuppin P, Rudant J, Constantinou P, Gastaldi-Ménager C, Rachas A, de Roquefeuil L, Maura G, Caillol H, Tajahmady A, Coste J, Gissot C, Weill A, Fagot-Campagna A (2017). Value of a national administrative database to guide public decisions: from the Système National D'Information Interrégimes de L'Assurance Maladie (SNIIRAM) to the Système National des Données de Santé (SNDS) in France. Rev Epidemiol Sante Publique.

[17] Bezin J, Duong M, Lassalle R, Droz C, Pariente A, Blin P, Moore N (2017). The national healthcare system claims databases in France, SNIIRAM and EGB: powerful tools for pharmacoepidemiology. Pharmacoepidemiol Drug Saf.

[18] Rahib D, Le Guen M, Lydié N (2016). Baromètre Santé 2016. Contraception. Quatre ans Après la Crise de la Pilule, les Évolutions se Poursuivent.

[19] Richard JP, Andler R, Gautier A, Guignard R, Leon C, Beck F (2016). Effects of using an overlapping dual-frame design on estimates of health behaviors: a French general population telephone survey. J Surv Stat Methodol.

[20] Soriano LC, Wallander MA, Andersson S, Filonenko A, Rodríguez LAG (2014). Use of long-acting reversible contraceptives in the UK from 2004 to 2010: analysis using The Health Improvement Network Database. Eur J Contracept Reprod Health Care.

[21] Cea-Soriano L, Rodríguez LAG, Machlitt A, Wallander MA (2014). Use of prescription contraceptive methods in the UK general population: a primary care study. BJOG.

[22] Furu K, Aares EB, Hjellvik V, Karlstad Ø (2021). Hormonal contraceptive use in Norway, 2006-2020, by contraceptive type, age and county: a nationwide register-based study. Nor J Epidemiol.

[23] Kristensen SI, Lidegaard Ø (2021). Hormonal contraceptive use in Denmark 2010-2019. Dan Med J.

[24] Lindh I, Skjeldestad FE, Gemzell-Danielsson K, Heikinheimo O, Hognert H, Milsom I, Lidegaard Ø (2017). Contraceptive use in the Nordic countries. Acta Obstet Gynecol Scand.

[25] Lundin C, Wikman A, Lampa E, Bixo M, Gemzell-Danielsson K, Wikman P, Ljung R, Poromaa IS (2022). There is no association between combined oral hormonal contraceptives and depression: a Swedish register-based cohort study. BJOG.

[26] Toffol E, Heikinheimo O, But A, Latvala A, Partonen T, Haukka J (2021). Population-level indicators associated with hormonal contraception use: a register-based matched case-control study. BMC Public Health.

[27] Furu K, Wettermark B, Andersen M, Martikainen JE, Almarsdottir AB, Sørensen HT (2010). The Nordic countries as a cohort for pharmacoepidemiological research. Basic Clin Pharmacol Toxicol.

[28] (2014). Data quality statement—NHS contraceptive services, 2013-14. Health & Social Care Information Centre.

[29] Partin MR, Malone M, Winnett M, Slater J, Bar-Cohen A, Caplan L (2003). The impact of survey nonresponse bias on conclusions drawn from a mammography intervention trial. J Clin Epidemiol.

[30] Morgan CR, Liu H (2017). The relationship between area deprivation and prescription of long-acting reversible contraception in women of reproductive age in Lothian, Scotland, UK. J Fam Plann Reprod Health Care.

[31] Tuppin P, Samson S, Colinot N, Gastaldi-Menager C, Fagot-Campagna A, Gissot C (2016). Health care use by free complementary health insurance coverage beneficiaries in France in 2012. Rev Epidemiol Sante Publique.

