# Supplementary material for: Contraceptive Use Measured in a National Population–Based Approach: Cross-Sectional Study of Administrative Versus Survey Data
Source: JMIR Public Health Surveill. 2024 Jul 22;10:e45030. doi: 10.2196/45030 (PMC11301111; doi:10.2196/45030)
Supplement: Multimedia Appendix 1 [file publichealth_v10i1e45030_app1.docx]

| **ATC code^a^** | **Contraceptive code** | **Label Name** | **Recommended duration of use** |
| --- | --- | --- | --- |
| G02BA03 | 3400927419478 | JAYDESS 13,5 MG DISP INTRA UTERIN 1 | 3 years |
|  | 3400930094754 | KYLEENA 19,5 MG DISP INTRA UTERIN 1 | 5 years |
|  | 3400933929282 | MIRENA 52 MG DISP INTRA UTERIN 1 | 5 years |
| G03AA05 | 3400930677667 | MILLI ANOVLAR CPR 21 | 1 month |
|  | 3400930677438 | MILLI ANOVLAR CPR 63 | 3 months |
|  | 3400931853596 | TRENTOVLANE CPR 21 | 1 month |
|  | 3400931853657 | TRENTOVLANE CPR 63 | 3 months |
|  | 3400932549139 | TRIELLA CPR 21 | 1 month |
|  | 3400932549368 | TRIELLA CPR 63 | 3 months |
| G03AA06 | 3400930996195 | STEDIRIL 0,5 MG/0,05 MG CPR 21 | 1 month |
|  | 3400930996256 | STEDIRIL 0,5 MG/0,05 MG CPR 63 | 3 months |
| G03AA07 | 3400931840640 | ADEPAL CPR 21 | 1 month |
|  | 3400931840879 | ADEPAL CPR 63 | 3 months |
|  | 3400930149164 | ASTERLUNA 100 MCG/20 MCG CPR 21 | 1 month |
|  | 3400930149171 | ASTERLUNA 100 MCG/20 MCG CPR 63 | 3 months |
|  | 3400930149140 | ASTERLUNA CONTINU 100 MCG/20 MCG CPR 0 | 1 month |
|  | 3400930149157 | ASTERLUNA CONTINU 100 MCG/20 MCG CPR 84 | 3 months |
|  | 3400926921200 | EFFILEVO 100 MCG/20 MCG CPR 21 | 1 month |
|  | 3400926921378 | EFFILEVO 100 MCG/20 MCG CPR 63 | 3 months |
|  | 3400926921439 | EFFILEVO CONTINU 100/20 MCG CPR 28 | 1 month |
|  | 3400926921668 | EFFILEVO CONTINU 100/20 MCG CPR 84 | 3 months |
|  | 3400922408378 | ETHINYL/LEVON.TVC 30/150 MCG CPR 21 | 1 month |
|  | 3400922408439 | ETHINYL/LEVON.TVC 30/150 MCG CPR 63 | 3 months |
|  | 3400938479263 | LEELOO 0,1 MG/0,02 MG CPR 21 | 1 month |
|  | 3400938479324 | LEELOO 0,1 MG/0,02 MG CPR 63 | 3 months |
|  | 3400930066690 | LEELOO CONTINU 100/20 MCG CPR 28 | 1 month |
|  | 3400930066713 | LEELOO CONTINU 100/20 MCG CPR 84 | 3 months |
|  | 3400928023308 | LEVONOR/ETHINYL BGA 0.15/0,03 MG CPR 21 | 1 month |
|  | 3400928023476 | LEVONOR/ETHINYL BGA 0.15/0,03 MG CPR 63 | 3 months |
|  | 3400930017531 | LEVONOR/ETHINYL BGA 100/20 MICROG CPR 21 | 1 month |
|  | 3400930017548 | LEVONOR/ETHINYL BGA 100/20 MICROG CPR 63 | 3 months |
|  | 3400930066737 | LEVONOR/ETHINYL BGACONT 100/20 MCG CP 84 | 3 months |
|  | 3400930033326 | LEVONOR/ETHINYL CRT 100/20 MICROG CPR 63 | 3 months |
|  | 3400927982552 | LEVONOR/ETHINYL EG 0.15/0,03 MG CPR 21 | 1 month |
|  | 3400927982613 | LEVONOR/ETHINYL EG 0.15/0,03 MG CPR 63 | 3 months |
|  | 3400927838392 | LEVONOR/ETHINYL EG 100/20 MICROG CPR 21 | 1 month |
|  | 3400927838453 | LEVONOR/ETHINYL EG 100/20 MICROG CPR 63 | 3 months |
|  | 3400927983733 | LEVONOR/ETHINYL MYP 0.15/0,03 MG CPR 21 | 1 month |
|  | 3400927983962 | LEVONOR/ETHINYL MYP 0.15/0,03 MG CPR 63 | 3 months |
|  | 3400928023186 | LEVONOR/ETHINYL SDZ 0.15/0,03 MG CPR 21 | 1 month |
|  | 3400928023247 | LEVONOR/ETHINYL SDZ 0.15/0,03 MG CPR 63 | 3 months |
|  | 3400927714283 | LEVONOR/ETHINYL SDZ 100/20 MICROG CPR 21 | 1 month |
|  | 3400927714344 | LEVONOR/ETHINYL SDZ 100/20 MICROG CPR 63 | 3 months |
|  | 3400927999291 | LEVONOR/ETHINYL ZEN 0.15/0,03 MG CPR 21 | 1 month |
|  | 3400927999352 | LEVONOR/ETHINYL ZEN 0.15/0,03 MG CPR 63 | 3 months |
|  | 3400927731723 | LEVONOR/ETHINYL ZEN 100/20 MICROG CPR 21 | 1 month |
|  | 3400927731891 | LEVONOR/ETHINYL ZEN 100/20 MICROG CPR 63 | 3 months |
|  | 3400930140567 | LOLISTREL CONTINU 100/20 MCG CPR 28 | 1 month |
|  | 3400930140574 | LOLISTREL CONTINU 100/20 MCG CPR 84 | 3 months |
|  | 3400927838163 | LOLISTREL GE 100MCG/20 MCG CPR 21 | 1 month |
|  | 3400927838224 | LOLISTREL GE 100MCG/20 MCG CPR 63 | 3 months |
|  | 3400922033396 | LOVAPHARM 30/150 MCG CPR 21 | 1 month |
|  | 3400922033457 | LOVAPHARM 30/150 MCG CPR 63 | 3 months |
|  | 3400939102344 | LOVAVULO 20 MICROG/100 MICROG CPR PELL 21 | 1 month |
|  | 3400939108087 | LOVAVULO 20 MICROG/100 MICROG CPR PELL 63 | 3 months |
|  | 3400935673169 | LUDEAL CPR 21 | 1 month |
|  | 3400935673220 | LUDEAL CPR 63 | 3 months |
|  | 3400927982323 | MILEVONI 0.15/0,03 MG CPR 21 | 1 month |
|  | 3400927982491 | MILEVONI 0.15/0,03 MG CPR 63 | 3 months |
|  | 3400927892332 | MILEVONI 100/20 MICROG CPR 21 | 1 month |
|  | 3400927892561 | MILEVONI 100/20 MICROG CPR 63 | 3 months |
|  | 3400931823902 | MINIDRIL 0,15/0,03 MG CPR 21 | 1 month |
|  | 3400931824091 | MINIDRIL 0,15/0,03 MG CPR 63 | 3 months |
|  | 3400926752514 | OPTIDRIL 30/150 MCG CPR 28 | 1 month |
|  | 3400926752682 | OPTIDRIL 30/150 MCG CPR 84 | 3 months |
|  | 3400922178967 | OPTILOVA 20/100 MCG CPR 28 | 1 month |
|  | 3400922179049 | OPTILOVA 20/100 MCG CPR 84 | 3 months |
|  | 3400939181066 | PACILIA CPR 21 | 1 month |
|  | 3400939181127 | PACILIA CPR 63 | 3 months |
|  | 3400949113361 | QIADE GE 150/30 MCG CPR 21 | 1 month |
|  | 3400949113422 | QIADE GE 150/30 MCG CPR 63 | 3 months |
| G03AA09 | 3400939720579 | DESOBEL 150 MCG/20 MCG G¿ CPR 21 | 1 month |
|  | 3400939720630 | DESOBEL 150 MCG/20 MCG G¿ CPR 63 | 3 months |
|  | 3400939721231 | DESOBEL 150 MCG/30 MCG G¿ CPR 21 | 1 month |
|  | 3400939721460 | DESOBEL 150 MCG/30 MCG G¿ CPR 63 | 3 months |
|  | 3400935866219 | DESOGESTREL/ETHINYLESTRADIOL BIOGARAN 150/20 CPR 21 | 1 month |
|  | 3400935866387 | DESOGESTREL/ETHINYLESTRADIOL BIOGARAN 150/20 CPR 63 | 3 months |
|  | 3400935866448 | DESOGESTREL/ETHINYLESTRADIOL BIOGARAN 150/30 CPR 21 | 1 month |
|  | 3400935866509 | DESOGESTREL/ETHINYLESTRADIOL BIOGARAN 150/30 CPR 63 | 3 months |
|  | 3400939720869 | DESOGESTREL/ETHINYLESTRADIOL ELKA 150 MCG/20 MCG CPR 21 | 1 month |
|  | 3400939720920 | DESOGESTREL/ETHINYLESTRADIOL ELKA 150 MCG/20 MCG CPR 63 | 3 months |
|  | 3400939721989 | DESOGESTREL/ETHINYLESTRADIOL ELKA 150 MCG/30 MCG CPR 21 | 1 month |
|  | 3400939722061 | DESOGESTREL/ETHINYLESTRADIOL ELKA 150 MCG/30 MCG CPR 63 | 3 months |
|  | 3400935037183 | DESOGESTREL/ETHINYLESTRADIOL QUILL 150 MCG/20 MCG CPR 21 | 1 month |
|  | 3400935037244 | DESOGESTREL/ETHINYLESTRADIOL QUILL 150 MCG/20 MCG CPR 63 | 3 months |
|  | 3400935037763 | DESOGESTREL/ETHINYLESTRADIOL QUILL 150 MCG/30 MCG CPR 21 | 1 month |
|  | 3400935037824 | DESOGESTREL/ETHINYLESTRADIOL QUILL 150 MCG/30 MCG CPR 63 | 3 months |
|  | 3400933853839 | VARNOLINE CONTINU CPR 28 | 1 month |
|  | 3400933854089 | VARNOLINE CONTINU CPR 84 | 3 months |
| G03AA10 | 3400937596138 | CARLIN 75 MCG/20 MCG G¿ CPR 21 | 1 month |
|  | 3400937596367 | CARLIN 75 MCG/20 MCG G¿ CPR 63 | 3 months |
|  | 3400937595476 | CARLIN 75 MCG/30 MCG G¿ CPR 21 | 1 month |
|  | 3400937595537 | CARLIN 75 MCG/30 MCG G¿ CPR 63 | 3 months |
|  | 3400937596428 | EFEZIAL 75 MCG/20 MCG G¿ CPR 21 | 1 month |
|  | 3400937596596 | EFEZIAL 75 MCG/20 MCG G¿ CPR 63 | 3 months |
|  | 3400937596886 | EFEZIAL 75 MCG/30 MCG G¿ CPR 21 | 1 month |
|  | 3400937596947 | EFEZIAL 75 MCG/30 MCG G¿ CPR 63 | 3 months |
|  | 3400937734417 | GESTODENE/ETHINYLESTRADIOL ARROW 75 MCG/20 MCG CPR 21 | 1 month |
|  | 3400937734585 | GESTODENE/ETHINYLESTRADIOL ARROW 75 MCG/20 MCG CPR 63 | 3 months |
|  | 3400937737029 | GESTODENE/ETHINYLESTRADIOL ARROW 75 MCG/30 MCG CPR 21 | 1 month |
|  | 3400937737197 | GESTODENE/ETHINYLESTRADIOL ARROW 75 MCG/30 MCG CPR 63 | 3 months |
|  | 3400937464383 | GESTODENE/ETHINYLESTRADIOL BIOGARAN 75 MCG/20 MCG CPR 21 | 1 month |
|  | 3400937464444 | GESTODENE/ETHINYLESTRADIOL BIOGARAN 75 MCG/20 MCG CPR 63 | 3 months |
|  | 3400937464505 | GESTODENE/ETHINYLESTRADIOL BIOGARAN 75 MCG/30 MCG CPR 21 | 1 month |
|  | 3400937464673 | GESTODENE/ETHINYLESTRADIOL BIOGARAN 75 MCG/30 MCG CPR 63 | 3 months |
|  | 3400937935500 | GESTODENE/ETHINYLESTRADIOL RANBAXY 75 MCG/20 MCG CPR 63 | 3 months |
|  | 3400937935159 | GESTODENE/ETHINYLESTRADIOL RANBAXY 75 MCG/30 MCG CPR 63 | 3 months |
|  | 3400937734646 | GESTODENE/ETHINYLESTRADIOL RATIOPHARM 75 MCG/20 MCG CPR 21 | 1 month |
|  | 3400937734707 | GESTODENE/ETHINYLESTRADIOL RATIOPHARM 75 MCG/20 MCG CPR 63 | 3 months |
|  | 3400937736886 | GESTODENE/ETHINYLESTRADIOL RATIOPHARM 75 MCG/30 MCG CPR 21 | 1 month |
|  | 3400937736947 | GESTODENE/ETHINYLESTRADIOL RATIOPHARM 75 MCG/30 MCG CPR 63 | 3 months |
|  | 3400937596657 | GESTODENE/ETHINYLESTRADIOL SANDOZ 75 MCG/20 MCG CPR 21 | 1 month |
|  | 3400937596718 | GESTODENE/ETHINYLESTRADIOL SANDOZ 75 MCG/20 MCG CPR 63 | 3 months |
|  | 3400937597029 | GESTODENE/ETHINYLESTRADIOL SANDOZ 75 MCG/30 MCG CPR 21 | 1 month |
|  | 3400937597197 | GESTODENE/ETHINYLESTRADIOL SANDOZ 75 MCG/30 MCG CPR 63 | 3 months |
|  | 3400938072464 | GESTODENE/ETHINYLESTRADIOL TEVA 75 MCG/20 MCG CPR 21 | 1 month |
|  | 3400938072525 | GESTODENE/ETHINYLESTRADIOL TEVA 75 MCG/20 MCG CPR 63 | 3 months |
|  | 3400938072006 | GESTODENE/ETHINYLESTRADIOL TEVA 75 MCG/30 MCG CPR 21 | 1 month |
|  | 3400938072174 | GESTODENE/ETHINYLESTRADIOL TEVA 75 MCG/30 MCG CPR 63 | 3 months |
|  | 3400937736657 | GESTODENE/ETHINYLESTRADIOL WINTHROP 75 MCG/20 MCG CPR 21 | 1 month |
|  | 3400937736718 | GESTODENE/ETHINYLESTRADIOL WINTHROP 75 MCG/20 MCG CPR 63 | 3 months |
|  | 3400937737258 | GESTODENE/ETHINYLESTRADIOL WINTHROP 75 MCG/30 MCG CPR 21 | 1 month |
|  | 3400937737319 | GESTODENE/ETHINYLESTRADIOL WINTHROP 75 MCG/30 MCG CPR 63 | 3 months |
| G03AB03 | 3400937700009 | AMARANCE CPR 63 | 3 months |
|  | 3400935884138 | DAILY CPR 21 | 1 month |
|  | 3400935884367 | DAILY CPR 63 | 3 months |
|  | 3400938999754 | EVANECIA CPR 21 | 1 month |
|  | 3400938999815 | EVANECIA CPR 63 | 3 months |
|  | 3400930081051 | TRINORDIOL CPR 21 | 1 month |
|  | 3400930081068 | TRINORDIOL CPR 63 | 3 months |
|  | 3400932807093 | TRINORDIOL CPR SUREMBALLE 21 | 1 month |
|  | 3400932807154 | TRINORDIOL CPR SUREMBALLE 63 | 3 months |
| G03AB04 | 3400932026326 | MINIPHASE (ACETATE DE NORETHISTERONE, ETHINYLESTRA | 1 month |
|  | 3400932026494 | MINIPHASE (ACETATE DE NORETHISTERONE, ETHINYLESTRA | 1 month |
| G03AC03 | 3400932200672 | MICROVAL 0,03 MG CPR 28 | 1 month |
|  | 3400932200733 | MICROVAL 0,03 MG CPR 84 | 3 months |
| G03AC08 | 3400935154439 | NEXPLANON 68 MG IMPLANT 1 | 3 years |
| G03AC09 | 3400922474144 | ANTIGONE 75 MCG CPR 28 | 1 month |
|  | 3400922474373 | ANTIGONE 75 MCG CPR 84 | 3 months |
|  | 3400941918308 | CLAREAL 75 MCG CPR 28 | 1 month |
|  | 3400941918476 | CLAREAL 75 MCG CPR 84 | 3 months |
|  | 3400926736484 | DESOGESTREL BGA 75 MCG CPR 28 | 1 month |
|  | 3400926736545 | DESOGESTREL BGA 75 MCG CPR 84 | 3 months |
|  | 3400930093535 | DESOGESTREL CRT 75 MCG CPR 84 | 3 months |
|  | 3400926954666 | DESOGESTREL EG 75 MCG CPR 28 | 1 month |
|  | 3400926954727 | DESOGESTREL EG 75 MCG CPR 84 | 3 months |
|  | 3400941918018 | DESOGESTREL MITHRA 75 MCG CPR 28 | 1 month |
|  | 3400941918186 | DESOGESTREL MITHRA 75 MCG CPR 84 | 3 months |
|  | 3400930145463 | DESOGESTREL MYLAN PHARMA 75 MCG CPR 0 | 1 month |
|  | 3400930145470 | DESOGESTREL MYLAN PHARMA 75 MCG CPR 84 | 3 months |
|  | 3400930055595 | DESOGESTREL SDZ 75 MCG CPR 28 | 1 month |
|  | 3400930055601 | DESOGESTREL SDZ 75 MCG CPR 84 | 3 months |
|  | 3400941912443 | DESOGESTREL ZEN 75 MCG CPR 28 | 1 month |
|  | 3400941912672 | DESOGESTREL ZEN 75 MCG CPR 84 | 3 months |
|  | 3400926808037 | DESOPOP 75 MCG CPR 28 | 1 month |
|  | 3400926808266 | DESOPOP 75 MCG CPR 84 | 3 months |
|  | 3400921661477 | ELFASETTE MYL 75 MCG CPR 28 | 1 month |
|  | 3400921661538 | ELFASETTE MYL 75 MCG CPR 84 | 3 months |
|  | 3400927421600 | LACTINETTE 75 MCG CPR 28 | 1 month |
|  | 3400927421778 | LACTINETTE 75 MCG CPR 84 | 3 months |
|  | 3400927406812 | OPTIMIZETTE 75 MCG CPR 28 | 1 month |
|  | 3400927406980 | OPTIMIZETTE 75 MCG CPR 84 | 3 months |
| G03DA02 | 3400933930004 | GESTORAL 10 MG (ACETATE DE MEDROXYPROGESTERONE) 1 | 1/2 month |
|  | 3400933930233 | PROVERA 10 MG CPR SEC 14 | 1/2 month |
| G03DB01 | 3400932192946 | DUPHASTON 10 MG CPR 10 | 1/2 month |
| G03DB03 | 3400931308614 | COLPRONE 5 MG CPR 20 | 1/2 month |
| G03DB04 | 3400936557246 | LUTENYL 3,75 MG CPR 14 | 1/2 month |
|  | 3400932661121 | LUTENYL 5 MG CPR 10 | 1/2 month |
|  | 3400938691870 | NOMEGESTROL ARW 5 MG CPR 10 | 1/2 month |
|  | 3400938173451 | NOMEGESTROL BGA 5 MG CPR 10 | 1/2 month |
|  | 3400938172799 | NOMEGESTROL EG 5 MG CPR 10 | 1/2 month |
|  | 3400936453708 | NOMEGESTROL MYL 5 MG CPR 10 | 1/2 month |
|  | 3400938323917 | NOMEGESTROL RTP 5 MG CPR 10 | 1/2 month |
|  | 3400939016207 | NOMEGESTROL SDZ 5 MG CPR 10 | 1/2 month |
|  | 3400938280241 | NOMEGESTROL TVC 5 MG CPR 10 | 1/2 month |
|  | 3400938692532 | NOMEGESTROL ZEN 5 MG CPR 10 | 1/2 month |
| G03DB05 | 3400931789451 | LUTIONEX 0,5 MG CPR 30 | 1 month |
| G03DB06 | 3400936647466 | CHLORMADINONE MYL 10 MG CPR 12 | 1/2 month |
|  | 3400936327986 | CHLORMADINONE MYL 5 MG CPR 10 | 1/2 month |
|  | 3400937352161 | CHLORMADINONE MYLAN GENERIQUES 10 MG CPR 12 | 1/2 month |
|  | 3400937352222 | CHLORMADINONE MYLAN GENERIQUES 5 MG 1 BOITE DE 10, | 1/2 month |
|  | 3400936328587 | CHLORMADINONE QUALIMED 5 MG CPR 10 | 1/2 month |
|  | 3400936526600 | CHLORMADINONE SANDOZ 2 MG CPR 10 | 1/2 month |
|  | 3400936647527 | CHLORMADINONE SDZ 10 MG CPR 12 | 1/2 month |
|  | 3400936560147 | CHLORMADINONE SDZ 5 MG CPR 10 | 1/2 month |
|  | 3400936647237 | CHLORMADINONE THERAMEX 10 MG CPR 12 | 1/2 month |
|  | 3400936667747 | CHLORMADINONE THERAMEX 5 MG CPR 10 | 1/2 month |
|  | 3400936561847 | CHLORMADINONE TVC 10 MG CPR 12 | 1/2 month |
|  | 3400936526020 | CHLORMADINONE TVC 2 MG CPR 10 | 1/2 month |
|  | 3400936526310 | CHLORMADINONE TVC 5 MG CPR 10 | 1/2 month |
|  | 3400933943301 | LUTERAN 10 MG CPR 12 | 1/2 month |
|  | 3400930633144 | LUTERAN 2 MG (ACETATE DE CHLORMADINONE) 1 BOITE DE | 1/2 month |
|  | 3400930633205 | LUTERAN 5 MG CPR 10 | 1/2 month |
| G03DB07 | 3400932491544 | SURGESTONE 0,125 MG CPR 10 | 1/2 month |
|  | 3400932491834 | SURGESTONE 0,25 MG CPR 10 | 1/2 month |
|  | 3400933133146 | SURGESTONE 0,5 MG CPR 10 | 1/2 month |
|  | 3400933642860 | SURGESTONE 0,5 MG CPR 12 | 1/2 month |
| N/A^b^ | 1120717 | OBJET CONTRACEPTIF, STERILET AVEC INSERTEUR, 7 MED, NT 380, SHORT OU STANDARD | 10 years |
|  | 1103848 | OBJET CONTRACEPTIF, STERILET AVEC INSERTEUR, 7 MED, TT 380 | 10 years |
|  | 1128370 | OBJET CONTRACEPTIF, STERILET AVEC INSERTEUR, 7 MED, UT N 380 STANDARD | 10 years |
|  | 1122283 | OBJET CONTRACEPTIF, STERILET AVEC INSERTEUR, 7 MED, UT S 380 SHORT | 10 years |
|  | 1158536 | OBJET CONTRACEPTIF, STERILET AVEC INSERTEUR, AU CUIVRE | 10 years |
|  | 6172819 | OBJET CONTRACEPTIF, STERILET AVEC INSERTEUR, AU CUIVRE.,HRA PHARMA | 10 years |
|  | 6186566 | OBJET CONTRACEPTIF, STERILET AVEC INSERTEUR, AU CUIVRE.,LABO 7 MED | 10 years |
|  | 6186572 | OBJET CONTRACEPTIF, STERILET AVEC INSERTEUR, AU CUIVRE.,LABO GYNEAS | 10 years |
|  | 6184840 | OBJET CONTRACEPTIF, STERILET AVEC INSERTEUR, AU CUIVRE.,LABORATOIRE CCD | 10 years |
|  | 1135890 | OBJET CONTRACEPTIF, STERILET AVEC INSERTEUR, CONTREL, GYNEFIX | 10 years |
|  | 1173062 | OBJET CONTRACEPTIF, STERILET AVEC INSERTEUR, JANSSEN-CILAG, GYNE T 200 | 10 years |
|  | 1187615 | OBJET CONTRACEPTIF, STERILET AVEC INSERTEUR, JANSSEN-CILAG, GYNE T 380 | 10 years |
|  | 1171407 | OBJET CONTRACEPTIF, STERILET AVEC INSERTEUR, MONA LISA NV, MONA LISA CU375 SL RO | 10 years |
|  | 1121125 | OBJET CONTRACEPTIF, STERILET AVEC INSERTEUR, MONA LISA NV, MONA LISA CU375-RO | 10 years |
|  | 1106752 | OBJET CONTRACEPTIF, STERILET AVEC INSERTEUR, MONA LISA NV, MONA LISA CUT-380A | 10 years |
|  | 1132519 | OBJET CONTRACEPTIF, STERILET AVEC INSERTEUR, MONA LISA NV, MONA LISA NT CU380 | 10 years |
|  | 1101938 | OBJET CONTRACEPTIF, STERILET AVEC INSERTEUR, MULTILAN, CU 375 SL | 10 years |
|  | 1152960 | OBJET CONTRACEPTIF, STERILET AVEC INSERTEUR, MULTILAN, CU 375 STANDARD | 10 years |
|  | 1134760 | OBJET CONTRACEPTIF, STERILET AVEC INSERTEUR, PRODIMED, GYNELLE 375 | 10 years |
|  | 1132531 | OBJET CONTRACEPTIF, STERILET AVEC INSERTEUR, SCHERING SA, NOVA T | 10 years |
|  | 1146770 | OBJET CONTRACEPTIF, STERILET AVEC INSERTEUR, SCHERING SA, NOVA T 380 | 10 years |
|  | 1125749 | OBJET CONTRACEPTIF, STERILET AVEC INSERTEUR, THERAMEX, SERTALIA | 10 years |
|  | 1120717 | OBJET CONTRACEPTIF, STERILET AVEC INSERTEUR, 7 MED, NT 380, SHORT OU STANDARD | 10 years |

**^a^** Codes of the Anatomical Therapeutic Chemical (ATC) classification system.

^b^ Not applicable: ATC not applicable because copper IUDs are categorized as medical devices, not as drugs.
