# Supplementary material for: Contraceptive Use Measured in a National Population–Based Approach: Cross-Sectional Study of Administrative Versus Survey Data
Source: JMIR Public Health Surveill. 2024 Jul 22;10:e45030. doi: 10.2196/45030 (PMC11301111; doi:10.2196/45030)
Supplement: Multimedia Appendix 2 [file publichealth_v10i1e45030_app2.docx]

In French health administrative data, prevalence is estimated taking into account non-reimbursed contraceptives and people who had no health care during the study period.

1. **Non-reimbursed contraceptives: third and fourth-generation combined oral contraceptives**

Third and fourth-generation combined oral contraceptives (COC) are not reimbursed in France and so are not included in French health administrative data. To compare administrative data with the most recent survey data, the prevalence estimated in health administrative data must be corrected in order to take into account third and fourth-generation COC. A correction factor was estimated based on another administrative database, a French pharmaceutical sales database (IQVIA Pharmatrend Micro). The IQVIA database exhaustively records all sales of reimbursed and non-reimbursed medicines in a sample of 11,400 drugstores nationwide, randomly selected after stratification on turnover, type of sales, location and catchment area. Between 1 January, 2016 and 31 December, 2016, the database recorded the sales of 42,123,458 units of COC (first, second, third and fourth-generation), a total which included 34,592,723 units of first and second-generation COC and 7,530,735 units of third and fourth-generation COC. Third and fourth-generation COC accounted for only 18% of all sales of these contraceptives, whereas first and second-generation COC accounted for 82%. The prevalence of all COC can be estimated as:

Prevalence of COC = Prevalence of first and second-generation COC × (1+0.18/0.82)

= Prevalence of first and second-generation COC × 1.2195

1. **People who had no health care during the study period**

The French health database includes information on reimbursed health care consumption. Thus, only people who had reimbursed health care during the study period can be observed. The prevalence estimated in the health administrative data has to be corrected in order to take into account people who received no reimbursed health care during the study period. A correction factor was estimated based on the referential table of the health insurance, that references all persons affiliated to the main French health insurance schemes, even those who have not received health care. This table showed that 96.949385% of women aged 15-49 years had received reimbursed health care.

Thus, prevalence can be estimated as:

Prevalence for all women = Prevalence for women who had received reimbursed health care during the study period × 0.96949385
