# Supplementary material for: Contraceptive Use Measured in a National Population–Based Approach: Cross-Sectional Study of Administrative Versus Survey Data
Source: JMIR Public Health Surveill. 2024 Jul 22;10:e45030. doi: 10.2196/45030 (PMC11301111; doi:10.2196/45030)
Supplement: Multimedia Appendix 3 [file publichealth_v10i1e45030_app3.docx]

| **Women aged 15-19 years** | | | | |
| --- | --- | --- | --- | --- |
|  | **Health administrative data** |  | **Survey data** | ***P* value^c^** |
|  | **(n=1,943,189)** |  | **(n=352)** |  |
|  | **% (95% CI)^a^** |  | **%^b^ (95% CI)** |  |
| **Implant** | 1.9 (1.9-1.9) |  | 1.5 (0.5-2.5) | =.04 |
| **IUD** | 0.6 (0.6-0.6) |  | 0.00 (0.00-0.00) |  |
| **Oral contraceptives** | 29.8 (29.8-29.9) |  | 25.9 (22.2-29.6) |  |
| **Not using contraception** | 32.3 (32.3-32.4) |  | 27.4 (23.6-31.1) |  |
| **Women aged 20-24 years** | | | | |
|  | **Health administrative data** |  | **Survey data** | ***P* value^c^** |
|  | **(n=1,923,509)** |  | **(n=488)** |  |
|  | **% (95% CI)^a^** |  | **%^b^ (95% CI)** |  |
| **Implant** | 4.6 (4.6-4.7) |  | 6.9 (4.9-9.0) | =.03 |
| **IUD** | 5.2 (5.1-5.2) |  | 3.8 (2.2-5.3) |  |
| **Oral contraceptives** | 44.2 (44.1-44.3) |  | 44.3 (40.2-48.3) |  |
| **Not using contraception** | 54.0 (53.9-54.1) |  | 55.0 (50.9-59.0) |  |

| **Women aged 25-29 years** | | | | |
| --- | --- | --- | --- | --- |
|  | **Health administrative data** |  | **Survey data** | ***P* value^c^** |
|  | **(n=2,093,726)** |  | **(n=588)** |  |
|  | **% (95% CI)^a^** |  | **%^b^ (95% CI)** |  |
| **Implant** | 4.2 (4.1-4.2) |  | 3.8 (2.3-5.3) | =.12 |
| **IUD** | 12.9 (12.9-13.0) |  | 15.1 (12.4-17.8) |  |
| **Oral contraceptives** | 34.1 (34.0-34.2) |  | 36.3 (32.6-40.0) |  |
| **Not using contraception** | 51.2 (51.1-51.2) |  | 55.2(51.4-59.0) |  |
| **Women aged 30-34 years** | | | | |
|  | **Health administrative data** |  | **Survey data** | ***P* value^c^** |
|  | **(n=2,173,256)** |  | **(n=647)** |  |
|  | **% (95% CI)^a^** |  | **%^b^ (95% CI)** |  |
| **Implant** | 3.6 (3.6-3.6) |  | 2.9 (1.6-4.2) | =.24 |
| **IUD** | 22.1 (22.0-22.1) |  | 24.4 (21.0-27.7) |  |
| **Oral contraceptives** | 26.6 (26.5-26.6) |  | 28.0 (24.5-31.5) |  |
| **Not using contraception** | 52.2 (52.2-52.3) |  | 55.3 (51.4-59.1) |  |
| **Women aged 35-39 years** | | | | |
|  | **Health administrative data** |  | **Survey data** | ***P* value^c^** |
|  | **(n=2,185,765)** |  | **(n=694)** |  |
|  | **% (95% CI)^a^** |  | **%^b^ (95% CI)** |  |
| **Implant** | 3.1 (3.1-3.2) |  | 2.0 (0.9-3.0) | =.19 |
| **IUD** | 28.1 (28.0-28.1) |  | 28.7 (25.2-32.2) |  |
| **Oral contraceptives** | 22.0 (21.9-22.0) |  | 24.2 (20.8-27.5) |  |
| **Not using contraception** | 53.2 (53.1-53.3) |  | 54.8 (51.0-58.7) |  |

| **Women aged 40-44 years** | | | | |
| --- | --- | --- | --- | --- |
|  | **Health administrative data** |  | **Survey data** | ***P* value^c^** |
|  | **(n=2,196,344)** |  | **(n=741)** |  |
|  | **% (95% CI)^a^** |  | **%^b^ (95% CI)** |  |
| **Implant** | 2.4 (2.3-2.4) |  | 3.2 (2.0-4.5) | =.003 |
| **IUD** | 28.2 (28.1-28.2) |  | 31.2 (27.9-34.6) |  |
| **Oral contraceptives** | 18.8 (18.8-18.9) |  | 21.5 (18.6-24.5) |  |
| **Not using contraception** | 49.3 (49.3-49.4) |  | 56.0 (52.4-59.6) |  |
| **Women aged 45-49 years** | | | | |
|  | **Health administrative data** |  | **Survey data** | ***P* value^c^** |
|  | **(n=2,254,464)** |  | **(n=775)** |  |
|  | **% (95% CI)^a^** |  | **%^b^ (95% CI)** |  |
| **Implant** | 1.5 (1.5-1.5) |  | 2.5 (1.3-3.6) | =.003 |
| **IUD** | 23.5 (23.4-23.5) |  | 27.7 (24.3-31.0) |  |
| **Oral contraceptives** | 15.7 (15.7-15.8) |  | 16.4 (13.7-19.2) |  |
| **Not using contraception** | 40.7 (40.6-40.7) |  | 46.6 (42.8-50.3) |  |

^a^CI Confidence interval

^b^ Weighted percentage

^c^ The values given are *P* values of the χ2 test, except for women aged 15-19 years where numbers were too small for use of this test. For this age group we used Fisher's exact test.
